# Effects of an ergothioneine-rich *Pleurotus* sp. on skin moisturizing functions and facial conditions: a randomized, double-blind, placebo-controlled trial

**DOI:** 10.3389/fmed.2024.1396783

**Published:** 2024-06-03

**Authors:** Motoki Hanayama, Koichiro Mori, Takahiro Ishimoto, Yukio Kato, Junya Kawai

**Affiliations:** ^1^Mushroom Research Laboratory, Hokuto Corporation, Nagano, Japan; ^2^Faculty of Pharmacy, Institute of Medical, Pharmaceutical and Health Sciences, Kanazawa University, Kanazawa, Japan

**Keywords:** *Pleurotus* species, ergothioneine, skin moisture, transepidermal water loss, wrinkle, texture, UV spot, pore

## Abstract

**Background:**

L-ergothioneine (EGT), an antioxidative and anti-inflammatory amino acid, is abundant in various mushroom fruiting bodies. Meanwhile, the effects of EGT-containing mushrooms on human skin are unknown. This study investigated the effects of oral ingestion of a novel EGT-rich strain of *Pleurotus* species (hiratake) on skin conditions in humans.

**Methods:**

We conducted a 12-week, randomized, double-blind, placebo-controlled, parallel-group trial to evaluate skin moisturizing functions and facial conditions in 80 healthy women who were randomly assigned to either a group that was supplemented with hiratake tablets containing 25 mg of EGT/day or a placebo group. Skin moisture content, transepidermal water loss (TEWL), and facial scores (VISIA scores) were measured at baseline, 8 weeks, and 12 weeks of supplementation.

**Results:**

At 8 weeks, the skin moisture content was significantly higher on the temple in the hiratake group than in the placebo group. The hiratake group also exhibited a significant increase in skin moisture content on the arm at 8 and 12 weeks compared with baseline. At 12 weeks, wrinkle and texture scores were significantly better in the hiratake group than in the placebo group, and plasma EGT concentrations in the hiratake group were 4.7-fold higher than baseline (from 3.4 to 15.9 μM). Furthermore, EGT concentrations in plasma were significantly correlated with improvements in skin moisture content and TEWL on the arm, implying that these skin moisturizing benefits could be partly attributed to EGT. A stratified analysis of participants with a low baseline plasma EGT concentration (< 3.3 μM) revealed that skin moisture content on the temple was significantly higher at 8 and 12 weeks, and skin moisture content on the arm at 12 weeks tended to be higher (*p* = 0.074), in the hiratake group than in the placebo group. These findings suggested that oral ingestion of EGT-rich hiratake can improve skin moisturizing functions.

**Conclusion:**

EGT-rich hiratake may help maintain skin conditions in healthy women, and EGT may play a role in these beneficial effects.

## Introduction

The skin, the largest organ in the body, is important for preventing excess water loss from the body and protecting against external physical, chemical, and biologic threats (1). Defective skin barrier function causes a condition known as dry skin, in which the surface may be rough, scaly, and flaky; these surface features are often accompanied by itching, burning, stinging, and a sensation of tightness (2). Most skin impairments are caused by extrinsic factors such as ultraviolet (UV) rays and air pollution, as well as intrinsic factors including oxidative stress and a decline in defense mechanisms (3). These factors play key roles in skin aging and damage (4). UV exposure and oxidative metabolism within the skin lead to the production of reactive oxygen species (ROS), which eventually cause skin aging (5–7). Therefore, mitigation of oxidative stress is necessary to maintain skin health.

L-ergothioneine (EGT) a food-derived antioxidant predominantly found in edible mushrooms (8). Plasma EGT concentrations have been correlated with decreased risks of mild cognitive impairment (9, 10), frailty (10), cardiovascular disease, and mortality (11). After oral ingestion, EGT is distributed across various tissues via its specific transporter, OCTN1/SL22A4, which is ubiquitously expressed throughout the body (12). In humans, orally administered EGT remains in plasma and whole blood for more than 4 weeks (13), partly because it undergoes minimal metabolism and its urinary excretion is reduced by OCTN1-mediated renal reabsorption (12, 14). Within the skin, OCTN1 is expressed particularly in the epidermis, where EGT is also abundantly distributed (15). Oral administration of *Coprinus comatus*, an EGT-containing edible mushroom, can inhibit UVB-induced DNA halogenation (an indicator of inflammation) in murine skin (16). EGT also protects UVB-irradiated keratinocytes, possibly by suppressing ROS and pro-inflammatory cytokines (17, 18). However, it is unclear whether EGT consumption can improve skin conditions in humans.

The fruiting bodies of a novel strain of *Pleurotus* species (19), known as Shimofuri-Hiratake (hiratake) and commonly consumed in Japan, are rich in EGT (53.2 mg/100 g). Therefore, we hypothesized that daily hiratake intake could help to maintain skin health. In the present study, a randomized, double-blind, placebo-controlled, parallel-group trial was conducted to investigate the effects of daily hiratake consumption (25 mg of EGT/day) on skin moisturizing functions and facial conditions. Additionally, Pearson correlation and stratified analyses were performed to explore the relationships of EGT consumption with skin moisturizing functions.

## Materials and methods

### Ethics approval

The human rights of individuals participating in this study were protected throughout the study. The study was conducted in accordance with the Declaration of Helsinki and the Japanese Ethical Guidelines for Medical and Health Research Involving Human Subjects. Written informed consent was obtained from all participants. The study protocol was approved by the clinical trial ethics review committee of Chiyoda Paramedical Care Clinic (approval date: 21 October 2022) and is publicly registered at UMIN-CTR (trial number: UMIN000049702).

### Participants

Among 192 healthy women who completed baseline measurements, 80 participants were selected based on the following inclusion criteria: (i) healthy women aged 20 to 64 years at the time of providing informed consent; (ii) individuals aware of dry or rough skin; and (iii) individuals who understood the study procedures and provided written informed consent to participate prior to the study. The exclusion criteria were as follows: (i) consumption of foods for specified health uses, foods with functional claims, supplements, and/or health foods that could affect skin condition more than 2 times per week; (ii) consumption of drugs or quasi-drugs that could affect skin condition more than 2 times per week; (iii) excessive sunburn or risk of excessive sunburn (e.g., due to travel or events); (iv) plans to change cosmetics; (v) plans to begin a new skin care routine; (vi) skin diseases (e.g., atopic dermatitis); (vii) presence of a bruise and/or scar near measurement sites; (viii) use of specific treatments near measurement sites (e.g., electrical facial treatment, peeling, or laser therapy); (ix) excessive alcohol consumption; (x) consumption of mushrooms more than 4 days per week; (xi) consumption of liver more than 4 days per week; (xii) dislike of mushrooms; (xiii) rough skin caused by pollinosis; (xiv) aware of menstruation-related skin deterioration; (xv) irregular life style (e.g., night shift work); (xvi) enrollment in other clinical trials involving medicine or health foods from the start of this trial until 4 weeks after its completion; (xvii) plans to change the shape of eyelashes or eyebrows; (xviii) past/current medical history of severe cardiac, hepatic, renal, or digestive diseases; (xix) pregnancy, lactation, or plans to become pregnant; (xx) risk of medicine/food allergy; (xxi) donation of whole blood and/or blood components (200 mL) within 1 month prior to this trial; (xxii) donation of whole blood (400 mL) within 4 months prior to this trial; (xxiii) blood collection (800 mL) within the past 12 months, including this trial; and (xxiv) ineligibility for the study, as determined by the investigator.

### Study design

This randomized, double-blind, placebo-controlled, parallel-group trial enrolled Japanese individuals at Chiyoda Paramedical Care Clinic (Tokyo, Japan) from December 2022 to April 2023. To our knowledge, there are no available data regarding the distribution of EGT in the skin after oral ingestion, although daily EGT administration for 1 week led to maximal EGT concentrations in whole blood approximately 4 weeks later in humans (13). Therefore, we hypothesized that daily ingestion of EGT would also lead to a gradual increase in EGT concentrations within the skin. Based on previous data regarding skin turnover rates (20), we planned to measure skin parameters at baseline, 8 weeks, and 12 weeks during the dietary intervention period. Additionally, we collected blood samples at baseline and 12 weeks. The primary outcome measured was skin moisturizing function (skin moisture content and transepidermal water loss (TEWL) on the left temple and arm). Facial conditions were analyzed as the secondary efficacy outcome. The sample size was determined based on previous studies that identified significant differences in skin moisturizing functions after oral supplementation (21, 22). Eighty participants were randomly assigned to two groups according to age, skin moisture content, and TEWL. Randomization was performed in a confidential manner by the participant assignment manager. Group assignments were disclosed to the participants, investigators, and analysts after data fixation.

### Test and placebo foods

Hiratake tablets were used as the test food. One tablet of the test food was 240 mg, and each participant ingested 21 tablets daily (25 mg of EGT/day). In the placebo tablets, hiratake powder was replaced by glucose and caramel. Fresh fruiting bodies of hiratake were cultivated by Hokuto Corporation (Nagano, Japan), then processed into dried powder. The compositions and nutrient contents of the test and placebo foods are shown in Supplementary Tables 1A, B.

### Assessments of skin properties

At baseline, 8 weeks, and 12 weeks, participants were instructed to wash their faces and hands and to remain in a room at a temperature of 21 ± 1°C with a humidity level of 50 ± 5% for at least 30 min. After this acclimation period, skin moisturizing function was assessed on the left temple and on the left forearm. Skin moisture content was measured five times using a Corneometer CM825 (Courage + Khazaka Electronic GmbH, Cologne, Germany); after exclusion of the maximum and minimum values, the mean of the remaining values was calculated. TEWL from skin was measured with a Tewameter TM300 (Courage + Khazaka Electronic GmbH, Cologne, Germany). Facial scores regarding wrinkles, texture, spots, UV spots, and pores were measured using the VISIA Evolution and its internal software (Canfield Inc., Fairfield, NJ, USA).

### Measurement of plasma EGT concentrations

Blood samples were mixed with heparin sodium and centrifuged to separate the plasma. Each plasma sample was then mixed with eight volumes (v/v) of acetonitrile and an equal volume of 10 μM d9-ergothioneine (d_9_-EGT; internal standard) in water. These mixtures were centrifuged, and the supernatants were analyzed using a triple quadruple mass spectrometer with electrospray ionization coupled to a liquid chromatography system (LCMS-8040; Shimadzu, Kyoto, Japan). The EGT standard and d_9_-EGT were provided by Tetrahedron (Paris, France). Chromatography was performed via step-gradient elution (10 mM ammonium acetate, 0.1% formic acid in 5% H_2_O and 95% acetonitrile in the initial step; 10 mM ammonium acetate, 0.1% formic acid in 20% H_2_O and 80% acetonitrile in the final step) on an ACQUITY UPLC BEH Amide Column (130 Å, 1.7 μm, 2.1 × 150 mm; Waters Corporation, Milford, MA, USA) at 40°C. The multiple reaction monitoring settings were 230.00 to 127.10 m/z for EGT and 239.15 to 127.00 m/z for d_9_-EGT.

### Statistical analysis

Within-group changes from baseline were assessed using the Wilcoxon signed-rank test with Bonferroni correction. Between-group differences at each time point were evaluated using the Wilcoxon rank-sum test. Correlations were evaluated by Pearson correlation analysis. Statistical analyses were conducted using Microsoft Excel 2021 (Microsoft, Redmond, WA, USA) and IBM SPSS Statistics version 26 (IBM, Armonk, NY, USA). Differences with *p*-values < 0.05 were considered statistically significant.

## Results

### Participant characteristics

**FIGURE 1:**
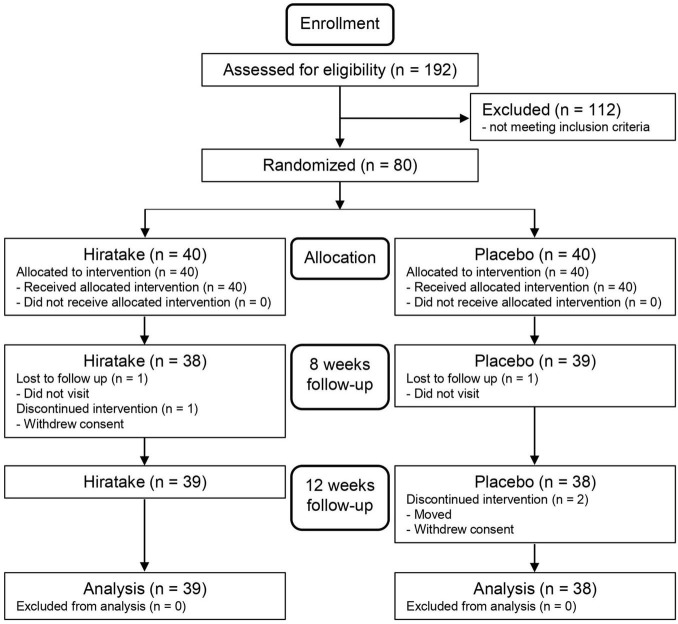
Flow diagram.

The flow diagram for the trial is presented in Figure 1. Table 1 shows the participants’ baseline characteristics. Overall, 77 participants (39 in the hiratake group and 38 in the placebo group) were included in the analysis. No participants withdrew from the study due to adverse effects related to the test foods.

**TABLE 1:** Baseline participant characteristics^a^.

	Hiratake (*n* = 39)	Placebo (*n* = 38)	*p*-value^b^
Sex	Female	Female	
Age (years)	48.0 ± 9.7	47.4 ± 9.9	0.882
**Moisture (a.u.)**			
Temple	60.3 ± 10.1	59.6 ± 9.3	0.621
Arm	25.7 ± 7.1	26.4 ± 7.2	0.811
**TEWL (g/m^2^ h)**			
Temple	13.6 ± 2.8	13.4 ± 2.8	0.647
Arm	8.83 ± 1.44	8.81 ± 1.47	0.899

^a^Values are presented as mean ± standard deviation. ^b^*p*-values for between-group differences were assessed by the Wilcoxon rank-sum test. a.u., arbitrary units; TEWL, transepidermal water loss.

### Improvements in skin moisturizing functions during hiratake supplementation

**FIGURE 2:**
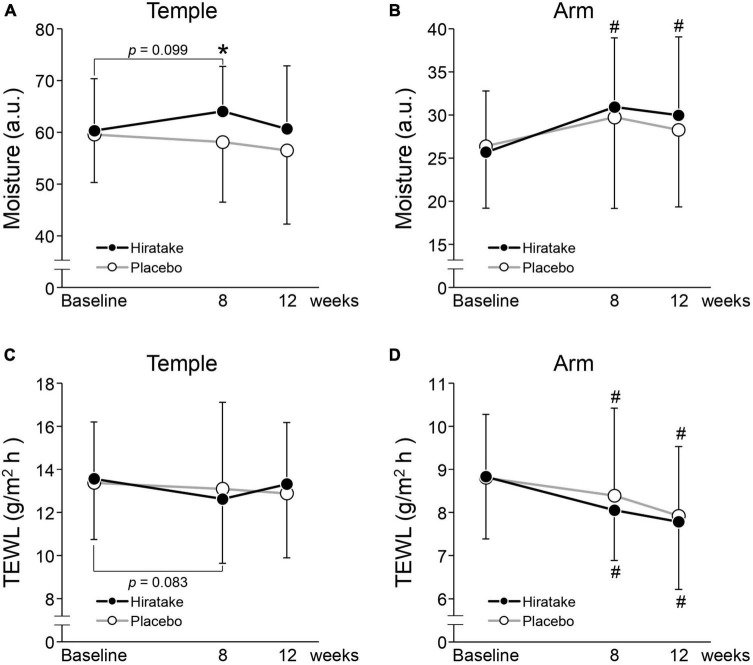
The effects of hiratake on skin moisturizing functions. Skin moisture content is shown for the **(A)** left temple and **(B)** arm, and TEWL is shown for the **(C)** left temple and **(D)** arm. Closed and open symbols indicate the hiratake (*n* = 39) and placebo (*n* = 38) groups, respectively. Values are presented as mean ± standard deviation. Significance was determined by the Wilcoxon rank-sum test for between-group differences (*p < 0.05) and by the Wilcoxon signed-rank test with Bonferroni correction for within-group changes compared with baseline (^#^*p* < 0.05). a.u., arbitrary units.

The effects of EGT-rich mushrooms on skin moisturizing functions were assessed by measuring the skin moisture content and TEWL on the left temple and arm. On the temple at 8 weeks, skin moisture content was significantly higher in the hiratake group than in the placebo group (Figure 2A). In the hiratake group, skin moisture content tended to increase (Figure 2A, *p* = 0.099) and TEWL tended to decrease (Figure 2C, *p* = 0.083) on the temple at 8 weeks compared with baseline. Furthermore, significant improvements in skin moisture content on the arm were observed in the hiratake group at both 8 and 12 weeks compared with baseline (Figure 2B), whereas no significant difference was observed in the placebo group. Although there was no significant difference in TEWL between the groups, a significant time-dependent decrease in TEWL on the arm was observed in both groups (Figure 2D).

### Effects of hiratake on facial conditions

Facial scores regarding wrinkles, texture, spots, UV spots, and pores were measured using the VISIA skin analysis system (Table 2). At 12 weeks, wrinkle and texture scores were significantly lower in the hiratake group than in the placebo group, suggesting improvements in wrinkles and texture upon ingestion of hiratake (Table 2). Additionally, the hiratake group exhibited significantly lower UV spot scores at 8 weeks than those at baseline (Table 2). The placebo group demonstrated significantly higher pore scores at 8 weeks and texture scores at 12 weeks than those scores at baseline, whereas the hiratake group did not exhibit significant changes in these scores (Table 2).

**TABLE 2:** Facial condition scores^a^.

VISIA values	Week	Hiratake (*n* = 39)	Placebo (*n* = 38)	Between-groups	Within-group *p*-values^c^	
				*p*-values^b^	Hiratake	Placebo
Wrinkle score	Baseline	10.1 ± 6.0	11.9 ± 7.0	0.271	–	–
	8	10.4 ± 6.7	12.3 ± 7.2	0.144	0.876	>1.000
	12	10.2 ± 6.2	13.0 ± 6.4	0.016	>1.000	0.142
Texture score	Baseline	5.61 ± 3.26	7.00 ± 4.32	0.21	–	–
	8	5.82 ± 3.75	7.21 ± 3.84	0.105	>1.000	>1.000
	12	5.82 ± 3.70	7.58 ± 4.00	0.034	>1.000	0.038
Spot score	Baseline	28.0 ± 7.0	29.1 ± 6.9	0.338	–	–
	8	27.5 ± 7.0	29.8 ± 6.2	0.122	0.893	>1.000
	12	28.0 ± 5.7	29.1 ± 5.9	0.299	>1.000	>1.000
UV spot score	Baseline	28.3 ± 4.9	28.1 ± 5.9	0.956	–	–
	8	27.4 ± 5.4	28.2 ± 5.9	0.595	0.034	0.646
	12	27.7 ± 5.1	27.8 ± 6.2	0.964	0.316	>1.000
Pore score	Baseline	15.2 ± 7.3	15.9 ± 8.6	0.895	–	–
	8	15.2 ± 8.1	17.4 ± 9.3	0.351	>1.000	0.041
	12	15.0 ± 7.2	16.9 ± 9.1	0.575	>1.000	0.290

^a^Values are presented as mean ± standard deviation. ^b^*p*-values for between-group differences were determined by Wilcoxon rank-sum test. ^c^*p*-values for within-group changes compared with baseline were determined by Wilcoxon signed-rank test with Bonferroni correction.

### EGT concentrations in plasma

**FIGURE 3:**
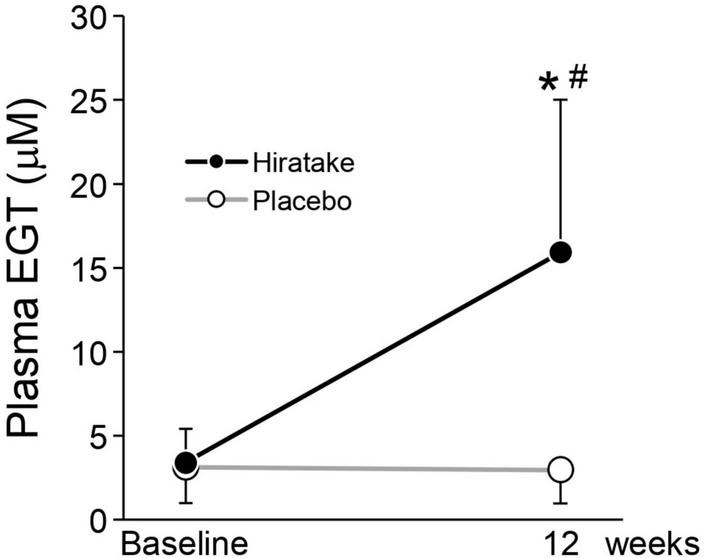
EGT concentrations in plasma. Closed and open symbols indicate the hiratake (*n* = 39) and placebo (*n* = 38) groups, respectively. Values are presented as mean ± standard deviation. Significance was determined by the Wilcoxon rank-sum test for between-group differences (*p < 0.05) and by the Wilcoxon signed-rank test for within-group changes compared with baseline (^#^*p* < 0.05).

There was no significant difference in the plasma EGT concentration at baseline between the two groups, and the mean concentration across all participants was 3.3 μM (Figure 3). At 12 weeks, the plasma EGT concentration was significantly higher in the hiratake group than in the placebo group, and a significant increase (from 3.4 μM at baseline to 15.9 μM at 12 weeks) was observed in the hiratake group but not the placebo group (Figure 3).

### Correlations of skin moisturizing functions with plasma EGT concentrations

**FIGURE 4:**
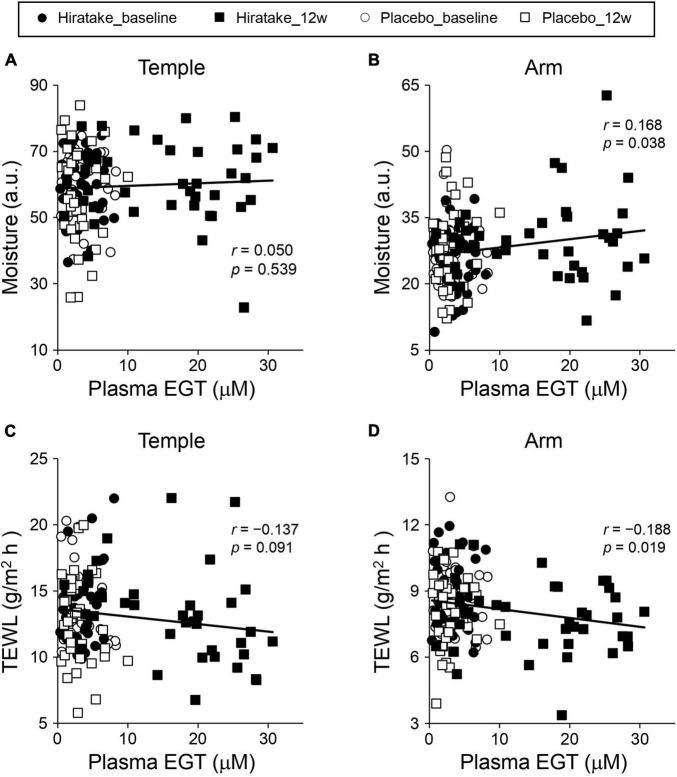
Scatter plots showing the correlation of plasma EGT concentration with each skin parameter (skin moisture content on the **(A)** left temple and **(B)** arm, and TEWL on the **(C)** left temple and **(D)** arm). The plots include all data regarding plasma EGT concentrations and skin parameters obtained at baseline and 12 weeks in the hiratake (*n* = 39) and placebo (*n* = 38) groups. Correlations were assessed by Pearson correlation analysis. *r*, correlation coefficient. a.u., arbitrary units.

To determine the contribution of EGT to the improvements in skin moisturizing functions during hiratake supplementation, we investigated correlations between skin parameters and plasma EGT concentrations by Pearson correlation analysis. Analyses of all data from both groups at baseline and 12 weeks revealed a significant positive correlation between skin moisture content on the arm and plasma EGT concentration (Figure 4B, *r* = 0.168, *p* = 0.038), whereas no significant correlation involving skin moisture content on the temple was observed (Figure 4A). Moreover, there was a significant negative correlation between TEWL on the arm and plasma EGT concentration (Figure 4D, *r* = −0.188, *p* = 0.019) and a tendency toward a negative correlation involving TEWL on the temple (Figure 4C, *r* = −0.137, *p* = 0.091).

### Skin moisture content among participants with a low baseline EGT concentration

**FIGURE 5:**
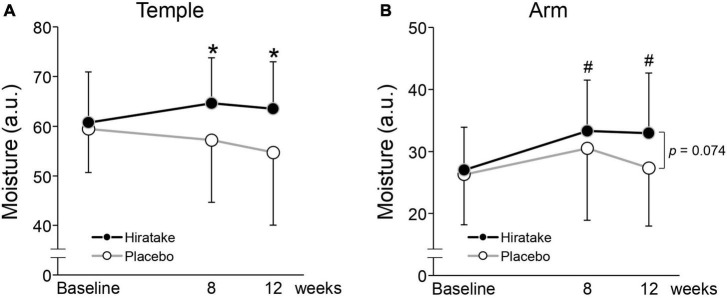
The effects of hiratake on skin moisture content among participants with a low baseline EGT concentration (< 3.3 μM). Skin moisture content is shown for the **(A)** left temple and **(B)** arm. Closed and open symbols indicate the hiratake (*n* = 19) and placebo (*n* = 25) groups, respectively. Values are presented as mean ± standard deviation. Significance was determined by the Wilcoxon rank-sum test for between-group differences (*p < 0.05) and by the Wilcoxon signed-rank test with Bonferroni correction for within-group changes compared with baseline (^#^*p* < 0.05). a.u., arbitrary units.

To evaluate the effects of hiratake in participants with limited variability in baseline EGT concentrations, a stratified analysis was conducted using data from participants with plasma EGT concentrations below the mean level at baseline (< 3.3 μM). The characteristics of participants with a low baseline EGT are presented in Supplementary Table 2. The analysis included 19 and 25 participants in the hiratake and placebo groups, respectively. Skin moisture content on the temple at 8 and 12 weeks was significantly higher in the hiratake group than in the placebo group (Figure 5A). Additionally, skin moisture content on the arm in the hiratake group was significantly higher at 8 and 12 weeks than at baseline (Figure 5B). Finally, skin moisture content on the arm at 12 weeks tended to be higher (*p* = 0.074) in the hiratake group than in the placebo group (Figure 5B).

### Adverse events

Throughout the trial, 24 mild adverse events were reported (11 and 13 in the hiratake and placebo groups, respectively). The principal investigator determined that none of these mild adverse events were related to the intake of either the hiratake or placebo tablets. No significant changes were observed in blood biochemistry analyses at 12 weeks (Supplementary Table 3). All participants who completed the study were included in the safety analysis set.

## Discussion

To our knowledge, this is the first study to investigate the effects of EGT-rich mushrooms on skin conditions in healthy women. Oral ingestion of hiratake improved skin moisturizing functions (Figure 2) and maintained or improved facial conditions (indicated by wrinkle, texture, UV spot, and pore scores; Table 2). The beneficial effects of hiratake on skin moisture content at 8 weeks (Figure 2A) are consistent with the previous finding that oral administration of *Flammulina velutipes*, which also contains components with beneficial effects on skin (e.g., EGT and glucosylceramide) (23, 24), led to improved skin moisture content (24). Moreover, significant differences between the two groups in wrinkle and texture scores at 12 weeks (Table 2), possibly caused by skin dryness (25), imply that hiratake can improve skin hydration at 8 weeks followed by skin structure at 12 weeks. However, there was no significant difference in skin moisture content between the two groups at 12 weeks (Figure 2A). The effects of hiratake at 12 weeks may have been masked by seasonal variations in test conditions such as UV intensity, temperature, and humidity.

In the present study, the ingestion of hiratake tablets containing EGT (25 mg/day) for 12 weeks caused plasma EGT concentrations to increase from 3.4 to 15.9 μM (Figure 3). This increase suggests that EGT from the hiratake tablets was efficiently absorbed within the range expected based on a previous report concerning oral ingestion of pure EGT (26). Therefore, mushrooms such as hiratake may be useful food sources for daily supplementation of EGT. Pearson correlation coefficients showed that improvements in skin moisturizing functions, assessed by measuring the skin moisture content and TEWL, during hiratake supplementation were weakly correlated with plasma EGT concentrations (Figures 4B, C, D), implying that EGT supplementation partly contributed to the observed improvements. Previous reports demonstrated that EGT is efficiently absorbed and retained in the human body (13, 26). In the present study, individual differences in plasma EGT concentration were observed among participants in the hiratake group: the concentrations ranged from 0.3 to 8.3 μM at baseline, and they ranged from 0.9 to 30.6 μM at 12 weeks in the hiratake group. This variation may be related to individual differences in daily food-derived EGT intake and EGT bioavailability during the study period, as well as the extensive variation in EGT accumulation within the skin (based on studies of skin isolated from adult donors) (15). Thus, it is difficult to completely exclude the effects of food-derived EGT consumed before the study on the observed skin parameters. A stratified analysis of participants with a low baseline plasma EGT concentration (< 3.3 μM) revealed significant improvements in skin moisture content on the temple at 8 and 12 weeks, along with a tendency for improvement on the arm at 12 weeks (*p* = 0.074), in the hiratake group compared with the placebo group (Figure 5). These results suggest that the efficacy of hiratake intake is higher among individuals with a lower baseline plasma concentration of EGT.

The ingestion of hiratake improved skin moisturizing functions on temple skin, which is likely exposed to natural sunlight (Figures 2A, C). UV radiation induces the formation of ROS such as superoxide radicals, hydroxyl radicals, and singlet oxygen (27). Meanwhile, EGT inhibits the production of these ROS (28–31), reduces UV-induced oxidative damage to cell components, and decreases apoptotic responses in keratinocytes (15). Additionally, in cultured UV-irradiated skin fibroblasts, EGT alleviates the decrease in collagen production (17, 32); increase in expression of matrix metalloproteinase-1 (17, 32), a major collagenolytic enzyme; and increase in gene expression of cellular communication network factor 1 (17), which is associated with diminished skin barrier function and reduced moisture (33). Therefore, EGT may have the potential to mitigate UV-induced oxidative stress and disruption of extracellular matrix homeostasis in the skin. The present study also showed a reduction in UV spot score among participants in the hiratake group. A clinical trial showed that topical application of EGT could decrease the melanin index in UVA-irradiated human skin by inhibiting tyrosinase activity within melanoma cells (34). Therefore, EGT-containing hiratake may also reduce UV spot scores by inhibiting tyrosinase activity.

Finally, hiratake intake improved skin moisture content compared with baseline on arm skin (Figure 2B), which is likely exposed to limited amounts of natural sunlight. EGT activates the Nrf2/Keep1 pathway, which upregulates antioxidant enzymes and intracellular glutathione, while enhancing procollagen expression, in fibroblasts not irradiated with UV (35). The antioxidative effect of EGT may reduce intrinsic oxidative stress and improve skin conditions. Furthermore, the observed improvement in skin moisture may be partly related to improved sleep quality, which affects skin conditions (36), based on a previous report that 4-week administration of EGT (20 mg/day) led to improved sleep quality (26).

The present study had some limitations. First, non-EGT components in hiratake may have effects on skin conditions. Therefore, the effects of pure EGT on skin conditions should be verified. Second, the sample sizes were small (hiratake, *n* = 39; placebo, *n* = 38); thus, the effects of hiratake on skin conditions should be verified in a larger study. Third, the effects of seasonal changes in skin could not be excluded. Future trials should be conducted during different seasons or locations with stable climates. Finally, the study only included female participants, so future trials should include male participants to enhance generalizability.

In conclusion, this study demonstrated that the ingestion of EGT-rich hiratake increases the plasma EGT concentration and improves skin conditions such as moisturizing functions, wrinkles, and texture. Our results suggest that the ingestion of EGT-rich mushrooms can have beneficial effects for individuals with skin conditions.

## Data availability statement

The raw data supporting the conclusions of this article will be made available by the authors, without undue reservation.

## Ethics statement

The studies involving humans were approved by the Clinical Trial Ethics Review Committee of Chiyoda Paramedical Care Clinic. The studies were conducted in accordance with the local legislation and institutional requirements. The participants provided their written informed consent to participate in this study.

## Author contributions

MH: Conceptualization, Formal analysis, Methodology, Writing – original draft, Writing – review & editing. KM: Conceptualization, Methodology, Writing – review & editing. TI: Writing – review & editing. YK: Writing – review & editing. JK: Conceptualization, Formal analysis, Methodology, Writing – original draft, Writing – review & editing.
