# Supplementary material for: Effects of an ergothioneine-rich Pleurotus sp. on skin moisturizing functions and facial conditions: a randomized, double-blind, placebo-controlled trial
Source: Front Med (Lausanne). 2024 Jun 3;11:1396783. doi: 10.3389/fmed.2024.1396783 (PMC11182000; doi:10.3389/fmed.2024.1396783)
Supplement: Supplementary file 1 [file Table_1.DOCX]

Supplementary Material

Effects of an Ergothioneine-rich *Pleurotus* sp. on Skin Moisturizing Functions and Facial Conditions: A Randomized, Double-blind, Placebo-controlled Trial

Motoki Hanayama^1*^, Koichiro Mori^1^, Takahiro Ishimoto^2^, Yukio Kato^2^, and Junya Kawai^1^

^1^*Mushroom Research Laboratory, Hokuto Corporation, Nagano, Japan*

^2^*Faculty of Pharmacy, Institute of Medical, Pharmaceutical and Health Sciences, Kanazawa University, Kanazawa, Japan*

*** Correspondence:**Motoki Hanayama

Address: Mushroom Research Laboratory, Hokuto Corporation, 800-8, Shimokomazawa, Nagano 381-0008, Japan.

Tel: +81-26-296-3211

E-mail: [motoki.hanayama@hokto-kinoko.co.jp](mailto:motoki.hanayama@hokto-kinoko.co.jp)

| **Supplementary Table 1A.** Compositions of the test and placebo foods (%) | | |  |
| --- | --- | --- | --- |
| Components | Hiratake | Placebo^a)^ |  |
| Hiratake powder | 79.4 | 0 |  |
| Glucose | 0 | 78.7 |  |
| Hydrogenated maltose starch syrup | 17.6 | 17.6 |  |
| Calcium stearate | 0.5 | 0.5 |  |
| Silicon dioxide | 1 | 1 |  |
| Hydroxypropyl cellulose | 1.5 | 1.5 |  |
| Caramel | 0 | 0.7 |  |
| ^a)^Placebo foods contained additional flavoring: 3.1×10^-4^% of 1-octen-3-ol and 1.5×10^-4^% of octanol | | | |
|  | | |  |
| **Supplementary Table 1B.** Nutrient compositions of the test and placebo foods (/100 g) | | |  |
| Components | Hiratake | Placebo |  |
| Energy (kcal) | 380 | 373 |  |
| Protein (g) | 26.4 | 0.2 |  |
| Carbohydrate (g) | 58.8 | 90.0 |  |
| Ash (g) | 6.8 | 1.1 |  |
| Fat (g) | 4.4 | 1.4 |  |
| Sodium (mg) | 8.9 | 8.9 |  |
|  |  |  |  |

**Supplementary Table 2.** EGT and skin parameters of participants with a low baseline EGT^a)^

| parameters | Week | Hiratake group  (n = 19) | Placebo group  (n = 25) | Between  groups  *p*-value^b)^ | Within-group *p*-value^c)^ | |
| --- | --- | --- | --- | --- | --- | --- |
|  |  |  |  |  | Hiratake | Placebo |
| Sex | Baseline | Female | Female | - | - | - |
| Age (years) | Baseline | 48.3 ± 9.2 | 46.4 ± 9.8 | 0.644 | - | - |
| EGT (μM) | Baseline | 1.72 ± 0.82 | 1.87 ± 0.75 | 0.586 | - | - |
|  | 12 | 16.0 ± 8.72 | 2.00 ± 1.00 | < 0.001 | < 0.001 | > 1.000 |
| Moisture (a.u.)  - Temple | Baseline | 60.8 ± 10.1 | 59.5 ± 8.8 | 0.496 | - | - |
|  | 8 | 64.7 ± 9.1 | 57.3 ± 12.6 | 0.045 | 0.312 | 0.844 |
|  | 12 | 63.6 ± 9.4 | 54.8 ± 14.7 | 0.032 | 0.748 | 0.202 |
| Moisture (a.u.)  - Arm | Baseline | 27.1 ± 6.9 | 26.4 ± 8.2 | 0.265 | - | - |
|  | 8 | 33.4 ± 8.2 | 30.6 ± 11.7 | 0.316 | 0.002 | 0.079 |
|  | 12 | 33.0 ± 9.7 | 27.4 ± 9.4 | 0.074 | 0.011 | 0.588 |
| TEWL (g/m^2^ h)  - Temple | Baseline | 13.2 ± 2.2 | 14.0 ± 3.1 | 0.619 | - | - |
|  | 8 | 13.1 ± 3.2 | 13.4 ± 4.3 | 0.971 | > 1.000 | > 1.000 |
|  | 12 | 14.0 ± 4.1 | 13.5 ± 3.5 | 0.907 | 0.752 | 0.778 |
| TEWL (g/m^2^ h)  - Arm | Baseline | 9.08 ± 1.51 | 8.93 ± 1.49 | 0.804 | - | - |
|  | 8 | 8.00 ± 1.33 | 8.60 ± 2.33 | 0.353 | > 1.000 | > 1.000 |
|  | 12 | 7.58 ± 1.96 | 7.69 ± 1.69 | 0.758 | > 1.000 | 0.678 |

^a)^Values are presented as mean ± standard deviation.

^b)^*p*-values for between-group differences were determined by Wilcoxon rank-sum test

^c)^*p*-values for within-group changes compared with baseline were determined by Wilcoxon signed-rank test with Bonferroni correction.

**Supplementary Table 3.** Blood biochemistry analyses of participants^a)^

| Clinical blood parameters | Week | Hiratake (n = 39) | Placebo^b)^ (n = 38) |
| --- | --- | --- | --- |
| White blood cell (number × 10^3^/μL) | Baseline | 5.46 ± 1.31 | 5.62 ± 1.23 |
|  | 12 | 5.54 ± 1.73 | 5.67 ± 1.59 |
| Red blood cell (number × 10^6^/μL) | Baseline | 4.41 ± 0.32 | 4.46 ± 0.33 |
|  | 12 | 4.42 ± 0.30 | 4.48 ± 0.32 |
| Hemoglobin (g/dL) | Baseline | 13.1 ± 0.8 | 13.3 ± 0.8 |
|  | 12 | 13.1 ± 0.9 | 13.4 ± 0.8 |
| Hematocrit (%) | Baseline | 40.8 ± 2.2 | 41.4 ± 2.2 |
|  | 12 | 41.3 ± 2.3 | 42.1 ± 2.2 |
| Platelet (number × 10^5^/μL) | Baseline | 2.70 ± 0.45 | 2.79 ± 0.66 |
|  | 12 | 2.59 ± 0.48 | 2.72 ± 0.60 |
| Total protein (g/dL) | Baseline | 7.15 ± 0.36 | 7.13 ± 0.36 |
|  | 12 | 7.07 ± 0.41 | 7.12 ± 0.30 |
| Albumin (g/dL) | Baseline | 4.45 ± 0.31 | 4.41 ± 0.24 |
|  | 12 | 4.44 ± 0.30 | 4.42 ± 0.22 |
| Total bilirubin (mg/L) | Baseline | 7.05 ± 2.58 | 6.79 ± 3.24 |
|  | 12 | 6.74 ± 2.48 | 7.08 ± 3.57 |
| ALP (U/L) | Baseline | 61.2 ± 19.0 | 61.4 ± 14.2 |
|  | 12 | 62.8 ± 18.6 | 64.2 ± 15.8 |
| LDH (U/L) | Baseline | 169 ± 29 | 164 ± 27 |
|  | 12 | 165 ± 27 | 164 ± 24 |
| AST (U/L) | Baseline | 21.5 ± 6.1 | 20.5 ± 4.5 |
|  | 12 | 21.1 ± 5.7 | 21.1 ± 4.3 |
| ALT (U/L) | Baseline | 16.9 ± 9.7 | 15.8 ± 7.9 |
|  | 12 | 16.3 ± 7.3 | 17.0 ± 7.7 |
| γ-GTP (U/L) | Baseline | 17.3 ± 7.0 | 18.1 ± 8.1 |
|  | 12 | 16.4 ± 6.7 | 19.2 ± 10.0 |
| CK (U/L) | Baseline | 94 ± 36 | 103 ± 75 |
|  | 12 | 97 ± 53 | 103 ± 60 |
| Total cholesterol (mg/dL) | Baseline | 208 ± 39 | 213 ± 35 |
|  | 12 | 206 ± 39 | 210 ± 35 |
| Triglycerides (mg/dL) | Baseline | 66.9 ± 27.9 | 68.8 ± 33.8 |
|  | 12 | 71.3 ± 35.7 | 72.6 ± 39.4 |
| HDL-C (mg/dL) | Baseline | 75.8 ± 15.9 | 74.0 ± 16.6 |
|  | 12 | 77.0 ± 16.1 | 73.9 ± 16.4 |
| LDL-C (mg/dL) | Baseline | 112 ± 28 | 118 ± 28 |
|  | 12 | 111 ± 32 | 118 ± 29 |
| Urea nitrogen (mg/dL) | Baseline | 11.7 ± 3.0 | 12.0 ± 2.8 |
|  | 12 | 11.7 ± 3.0 | 13.0 ± 3.5 |
| Creatinine (mg/L) | Baseline | 6.55 ± 0.79 | 6.44 ± 1.02 |
|  | 12 | 6.56 ± 0.80 | 6.67 ± 0.95 |
| Uric acid (mg/dL) | Baseline | 4.49 ± 0.90 | 4.29 ± 0.92 |
|  | 12 | 4.54 ± 1.13 | 4.50 ± 1.05 |
| Na (mEq/L) | Baseline | 140 ± 2 | 140 ± 2 |
|  | 12 | 141 ± 1 | 141 ± 2 |
| K (mEq/L) | Baseline | 4.27 ± 0.30 | 4.29 ± 0.28 |
|  | 12 | 4.19 ± 0.22 | 4.28 ± 0.30 |
| Cl (mEq/L) | Baseline | 104 ± 2 | 104 ± 2 |
|  | 12 | 104 ± 2 | 104 ± 2 |
| Ca (mg/dL) | Baseline | 9.34 ± 0.29 | 9.34 ± 0.36 |
|  | 12 | 9.19 ± 0.35 | 9.22 ± 0.38 |
| Glucose (mg/dL) | Baseline | 87.6 ± 6.8 | 90.0 ± 4.7 ** |
|  | 12 | 89.6 ± 8.0 | 89.3 ± 4.6 |
| HbA1c (%) | Baseline | 5.20 ± 0.27 | 5.21 ± 0.23 |
|  | 12 | 5.42 ± 0.30 | 5.41 ± 0.26 |

^a)^Values are presented as mean ± standard deviation.

^b)^Significance was determined by the Wilcoxon rank-sum test for between-group differences (** *p* < 0.01).
